# A Novel Dual-Step Nucleation Pathway in Crystalline Solids under Neutron Irradiation

**DOI:** 10.1038/s41598-017-18548-8

**Published:** 2018-01-08

**Authors:** Subhashish Meher, Isabella J. van Rooyen, Thomas M. Lillo

**Affiliations:** 1 Materials Science and Engineering Department, Idaho National Laboratory, Idaho Falls, ID 83415 USA; 2 Fuel Design and Development Department, Idaho National Laboratory, Idaho Falls, ID 83415 USA

## Abstract

Innovations in nanostructuring of inorganic crystalline solids are often limited by prerequisite critical nucleation energy and solute supersaturation for formation of a phase. This research provides direct evidence supporting the viability of an unconventional irradiation-induced nanostructuring process, via transmission electron microscopy, that circumvents these preconditions. Using polymorphic silicon carbide (SiC) as a prototype, a surprising two-step nucleation route is demonstrated through which nanoscale distribution of the second phase is achieved by reaction of solutes with neutron irradiation-induced precursors. In the first step, nanoscale α–SiC precipitates in a β–SiC matrix unexpectedly nucleate heterogeneously at structural defects. This occurs at significantly lower temperatures compared with the usual β→α transition temperature. Subsequently, α–SiC precipitate acts as a surrogate template for its structural and compositional transition into a fission product precipitate, palladium silicide. These discoveries provide a modern view of irradiation engineering in polymorphic ceramics for advanced applications.

## Introduction

Diverse aspects of science, ranging from organic to inorganic to mineral crystallization, have examined the nucleation process. Classical nucleation theory explains that the emergence of a stable cluster of a new, distinct second phase is dependent on overcoming critical nucleation energy^1^. It is well known that this activation energy is partially abated in metals and ceramics by heterogeneous precipitation of the second phase at structural defects such as grain boundaries^2^ or interphase interfaces^3^. Exploiting metallurgical principles for controlled distribution of intragranular nanoscale precipitates, particularly in ceramics, is an attractive prospect^4^.

Silicon carbide (SiC)—a polymorphic material—is known to exist in more than 250 polytypes^5^. Apart from electronic applications^6^, it is being considered as a radiation-tolerant material in nuclear fission and fusion reactors^7,8^ due to the isotropic nature of the 3 C β-SiC polytype. The irradiation of materials is widely observed to bring microstructural changes due to unexpected phase transformations^9–14^. Such irradiation engineering for nanostructured SiC can be useful for electronic or spintronic applications^15,16^.

This article describes a counterintuitive intragranular nucleation process in SiC that indirectly avoids the critical nucleation energy barrier. To the best of the research team’s knowledge, it cannot be explained completely by any known nucleation mechanism. This process provides a means for precipitating out a second phase in a rather incompliant matrix under neutron irradiation. This phase transition has been observed in neutron irradiated SiC utilized as a fission product barrier in TRISO (tristructural-isotropic) coated nuclear fuel particles^7^.

Although transport of fission products along grain boundaries of the β-SiC layer in TRISO coated fuel is most probable from a metallurgical perspective^17,18^, the intragranular nanoscale precipitation of fission products, mainly palladium silicide, has raised questions regarding their transport mechanisms^19,20^. The near homogenous distribution of these nanoscale Pd rich precipitates in β-SiC matrix^19,20^ has led to obvious conclusion based on classical nucleation theory to date that Pd interacts with β-SiC to form these precipitates. Hence, there have been numerous modeling effort to simulate the intragranular Pd transport in TRISO fuel by studying the interaction of Pd with β-SiC^21–23^. However, Gentile *et al*. has experimentally demonstrated that α-SiC is more susceptible than β-SiC to react with Pd^24^. The present novel dual-step nucleation mechanism of Pd silicide in SiC certainly gives light to these experimental-theoretical controversies of intragranular transport of fission products and has been demonstrated and explained in rest of the paper.

## Results and Discussion

**Figure 1:**
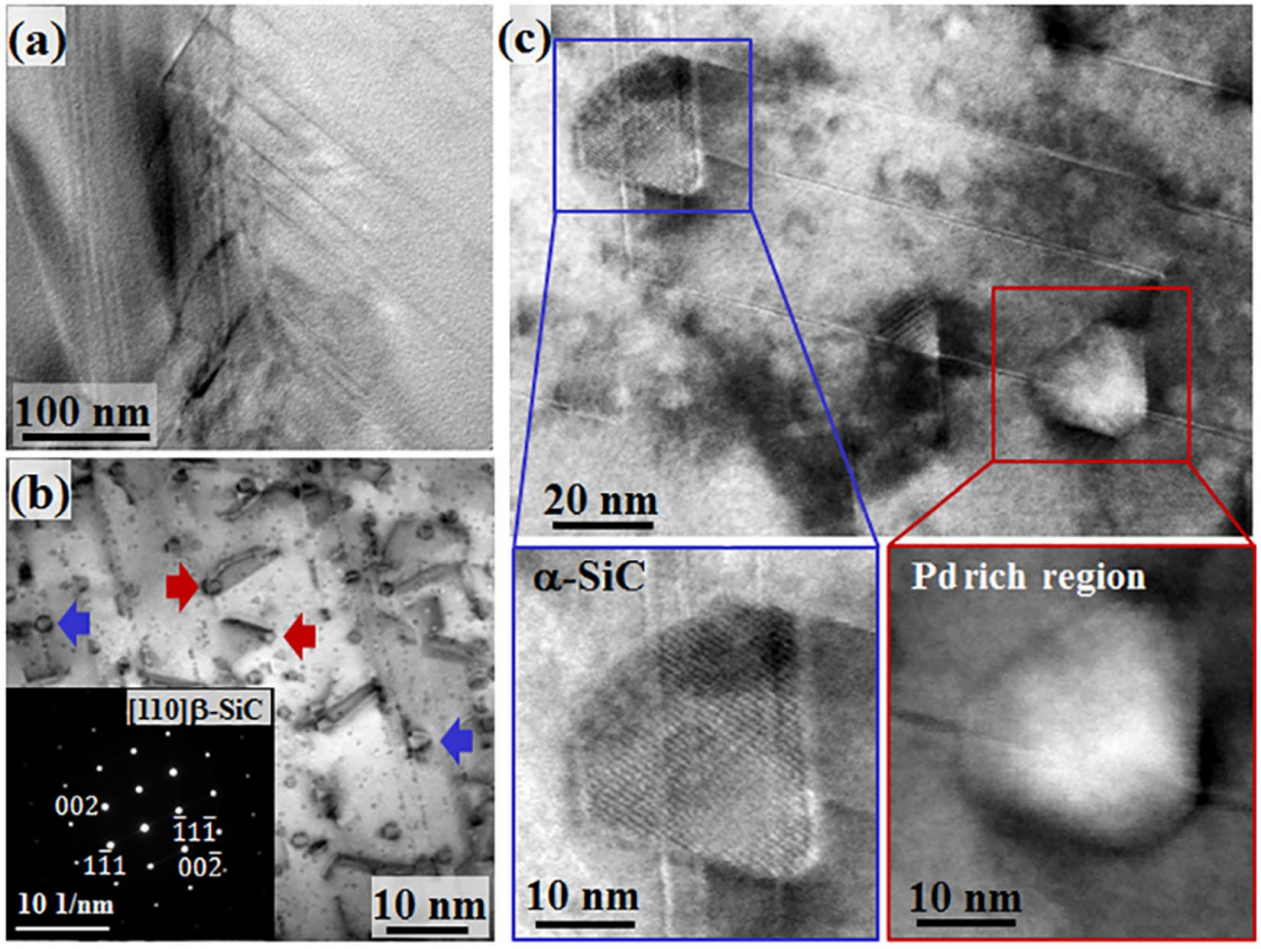
Neutron irradiation-induced microstructural changes in β-SiC layer of TRISO coated fuel particle. (**a**) A TEM bright field micrograph of an unirradiated β-SiC sample does not shows any Frank loop or polygonal precipitates. (**b**) A selected area diffraction (SAD) pattern of the matrix along [110] zone axis in the inset corresponds to cubic symmetry of the β-SiC. The SAD pattern indicates principal reflections of planes. A STEM micrograph of β-SiC, reveals both Frank loops and stacking faults induced due to irradiation at a fluence of 2.38 × 10^21^ n/m^2^ in a temperature range of 1,000–1,200 °C. Red arrows indicates the precipitates at the ends of Frank loops. Blue arrows indicate precipitates along stacking faults. (**c**) A STEM micrograph shows polygonal structures at structural defects with different chemical compositions. Energy dispersive spectrometry in TEM indicates a significant amount of Pd, along with Si and C, in the precipitate labelled by the red box. The precipitate labelled by the blue box contains only Si and C.

Transmission electron microscopy (TEM) of unirradiated SiC layer of TRISO fuel in Fig. 1(a) shows the presence of stacking faults but no apparent dislocation loops or precipitates are observed. TEM was consistently carried out here along the <011> direction of β-SiC as shown in the inset in Fig. 1(b). Irradiation-induced voids^25^ about 2–4 nm in diameter are visible in a scanning transmission electron microscopy (STEM) image in Fig. 1(b). Apart from these voids, polygonal-shaped precipitates with edge lengths of about 20–30 nm are mostly observed on Frank loops on {111} planes. The approximate volume fraction of these precipitates is less than one percent of the entire grain of β-SiC under study. Although these precipitates have similar size and morphology, some of them appear to possess strong mass contrast as shown in Fig. 1(c). Their chemical identities are revealed by the energy dispersive spectroscopy (EDS) analysis in TEM. While the precipitate indicated by the blue box in Fig. 1(d) did not contain any element other than Si and C, a significant amount of Pd (a fission product) was found along with Si and C in the precipitate indicated by the red box. The presence of a fission product other than Pd in these precipitates was not observed in this study, but the possibility of Pd-assisted transport of other elements cannot be ruled out^26^.

**Figure 2:**
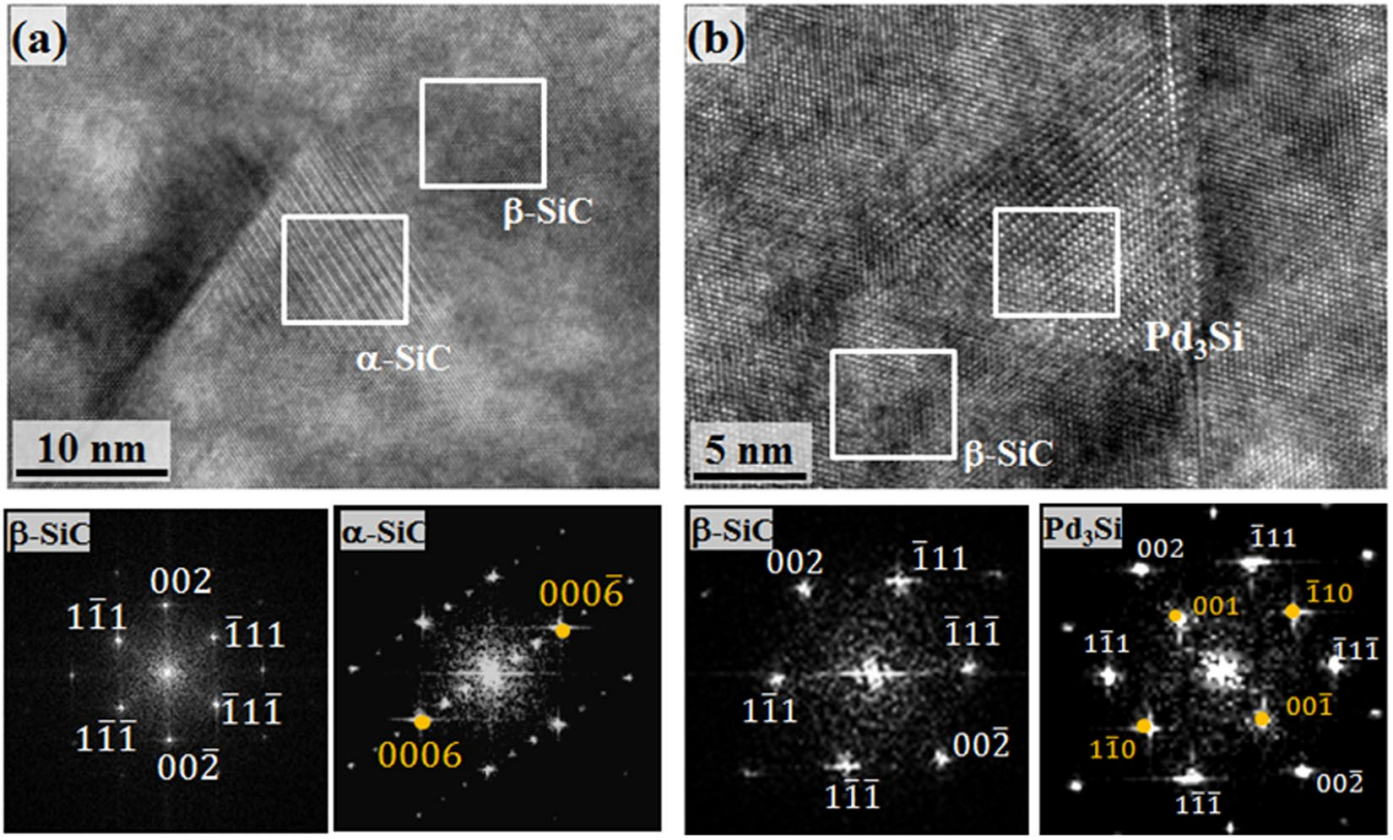
Crystallographic information of neutron irradiation-induced nanoscale precipitates. (**a**) A HRTEM micrograph along the [110] zone axis of β-SiC shows a α-SiC precipitate with one of its edges on a Frank loop. FFT of the β-SiC matrix shows only the principal diffraction spots of cubic symmetry. The FFT of the α-SiC shows the diffraction spots corresponding to the 6 H variant of SiC. A different orientation relationship between the α and β variants of SiC has been reported in Figure S1. (**b**) A HRTEM image along the [110] zone axis of β-SiC showing a Pd-rich precipitate that lies on a Frank loop. While the FFT of the β-SiC shows only the principal diffraction spots of a cubic structure, the FFT of the Pd-rich region shows the diffraction spots corresponding to L1_2_ ordered Pd_3_Si.

The structural analysis by high resolution (HR) TEM imaging further unveils the identities of these precipitates. Figure 2(a) shows a precipitate of Si and C only, with an average edge length of nearly 15 nm, in the β-SiC matrix at the edge of a Frank loop. While the fast Fourier transforms (FFT) of the matrix show only the principal reflections of the cubic β-SiC phase, the FFT of the precipitate corresponds to the 6 H variant of the α-SiC phase. The expected orientation relation between α-SiC and β-SiC, which is {111}β || {0001}α, is observed, since the lattice plane spacing d_(111)_ of β-SiC and d_0006_ of 6 H α-SiC are both 0.25 nm^27^. The usual transformation of β-SiC into α-SiC takes place at 2,000 °C or higher^27^. The presence of the α variant of SiC has been observed to nucleate at an irradiation temperature of 1,000–1,200 °C, which is nearly 50 percent lower than the transformation temperature^28^. This unusual behavior can be attributed to accelerated diffusion in Si and C that facilitates reconstructive transformation of β-SiC into α-SiC heterogeneously at linear and planar defects^28^. The structural defect assisted reconstructive transformation is expected to lower the nucleation energy barrier associated with α-SiC formation. Figures S1 and S2 shows the same transformation in SiC subjected to different experimental conditions. Similarly, HRTEM of another polygonal precipitate at a Frank loop is shown in Fig. 2(b). The FFT corresponding to the polygonal structure shows the superlattice diffraction spots, apart from the principal β-SiC spots, corresponding to a primitive L1_2_ cubic structure. It has been reported that Pd can react with Si within SiC to form ordered L1_2_ Pd_3_Si-type structures (space group Pnma)^21^. There are many other possibilities for structure and stoichiometry^21,29^. Previously, similar intergranular precipitates have been observed, but their structural identification has not been done^26^. Graphite, the byproduct of this solid state reaction, was not observed in this microscopic investigation.

**Figure 3:**
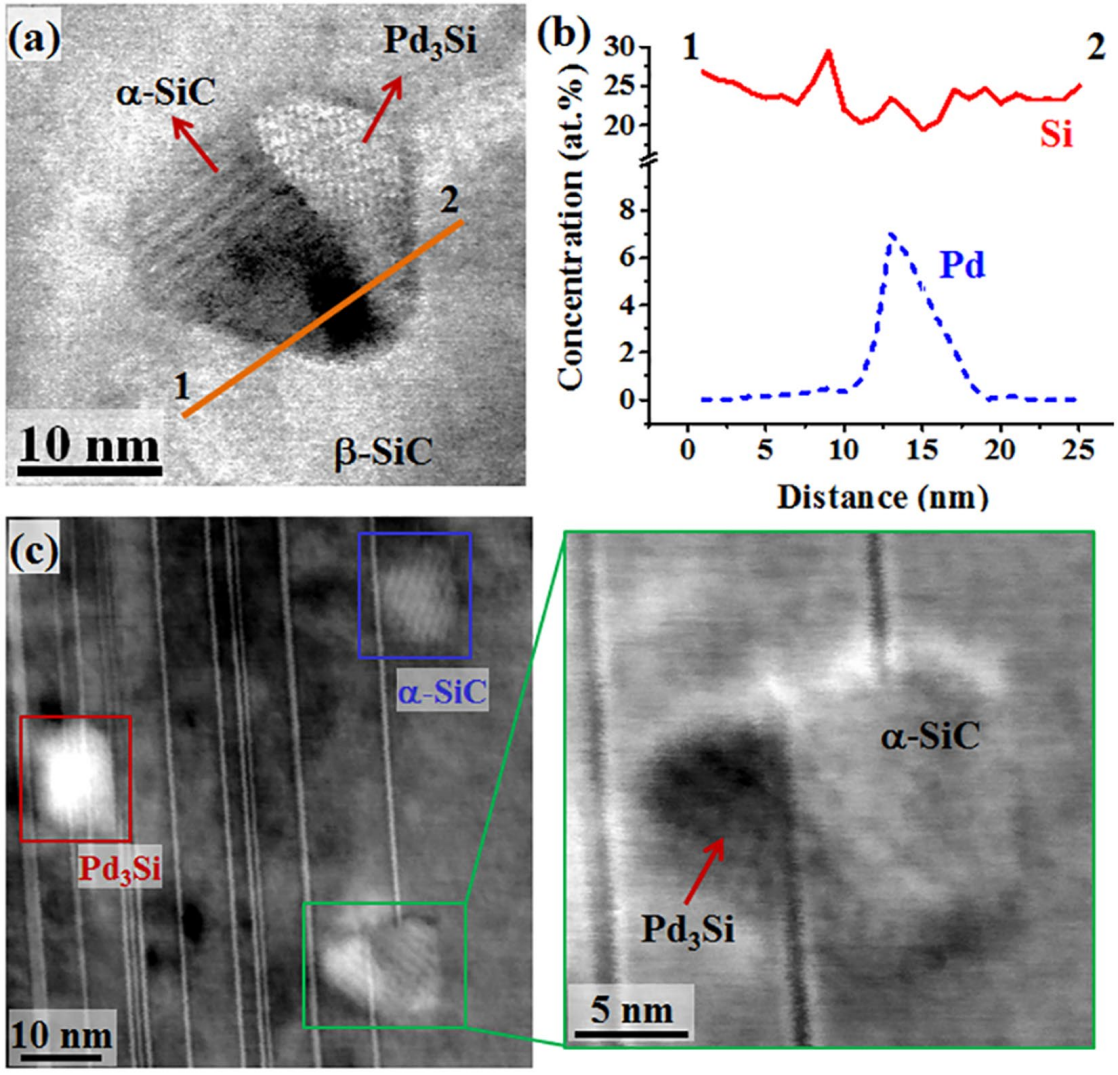
Direct observations of imprinting of Pd silicide into morphological templates of α-SiC precipitates. (**a**) A STEM micrograph along <110> of the β-SiC matrix clearly shows different crystallography and mass contrast within the hexagonal structure. (**b**) An EDS line profile, normal to the phase transition front, qualitatively shows a significant amount of Pd in the brighter part of the semi-transformed precipitate. (**c**) A STEM micrograph shows α-SiC, Pd silicide, and a semi-transformed region, labeled by blue, red, and green boxes, respectively. The α-SiC, Pd silicide lies across Frank loops. In the magnified image of the semi-transformed region, it appears that the Pd-rich and α-SiC regions are associated with two Frank loops terminating in opposite directions. To provide validated evidence of the two-step nucleation of Pd silicide, additional experiments on SiC fabricated by different processing parameters were performed and presented in Figure S2.

Unlike the isolated α-SiC and Pd-rich precipitates presented in Figs 1 and 2, an interesting feature in Fig. 3(a) is a nanoscale region that resides in β-SiC and has varied contrast within its own periphery. The image not only shows the contrast in mass of the two regions confined in a hexagonal periphery, but also the appearance of different crystallography. This precipitate appears to be isolated from any structural defects from the two-dimensional perspective of TEM, but the possibility of a defect out of the TEM foil cannot be denied. The linear chemical analysis along Line 1-2, normal to the phase transition front shown in Fig. 3(b), indicates that only the brighter region in the STEM image is chemically rich with Pd. A different region of β-SiC in Fig. 3(c) captures all three kinds of precipitates encountered in this study. In the magnified image of the semi-transformed region, indicated by the green box, it clearly appears that the Pd and α-SiC regions are associated with two Frank loops terminating in opposite directions. From this evidence, it appears that the diffusional transformation of α-SiC into Pd_3_Si is aided by the intragranular transport of Pd via linear or planar defects. However, there is possibility of Pd transport in the bulk β-SiC matrix^20,26^. These results lead to the intriguing puzzle of why Pd reacts with the α variant of SiC only. Interestingly, the presence of nanoscale α–SiC variant avails the readiness of Pd reaction with SiC as compared with that with a single phase, β-SiC^24^. It can be extrapolated that α-SiC precipitates potentially accelerated the Pd silicide formation. It is probably energetically expensive for Pd silicide to create new semi-coherent or incoherent interfaces with the β-SiC phase, together with the fact that it has a lower chemical affinity with β-SiC. This allows Pd_3_Si precipitates to be metamorphosed upon the surrogate α-SiC phase without movement of any phase boundary. Hence, this process uniquely circumvents the nucleation barrier by adopting the morphology of parent α-SiC rather than creating new interfaces with β-SiC. Also, the accelerated solute diffusion along structural defects does not require a conventional solute supersaturation for second phase precipitation. Though only one type of Pd silicide was found here, there is the possibility of formation of other types depending upon the processing conditions^21,29^.

**Figure 4:**
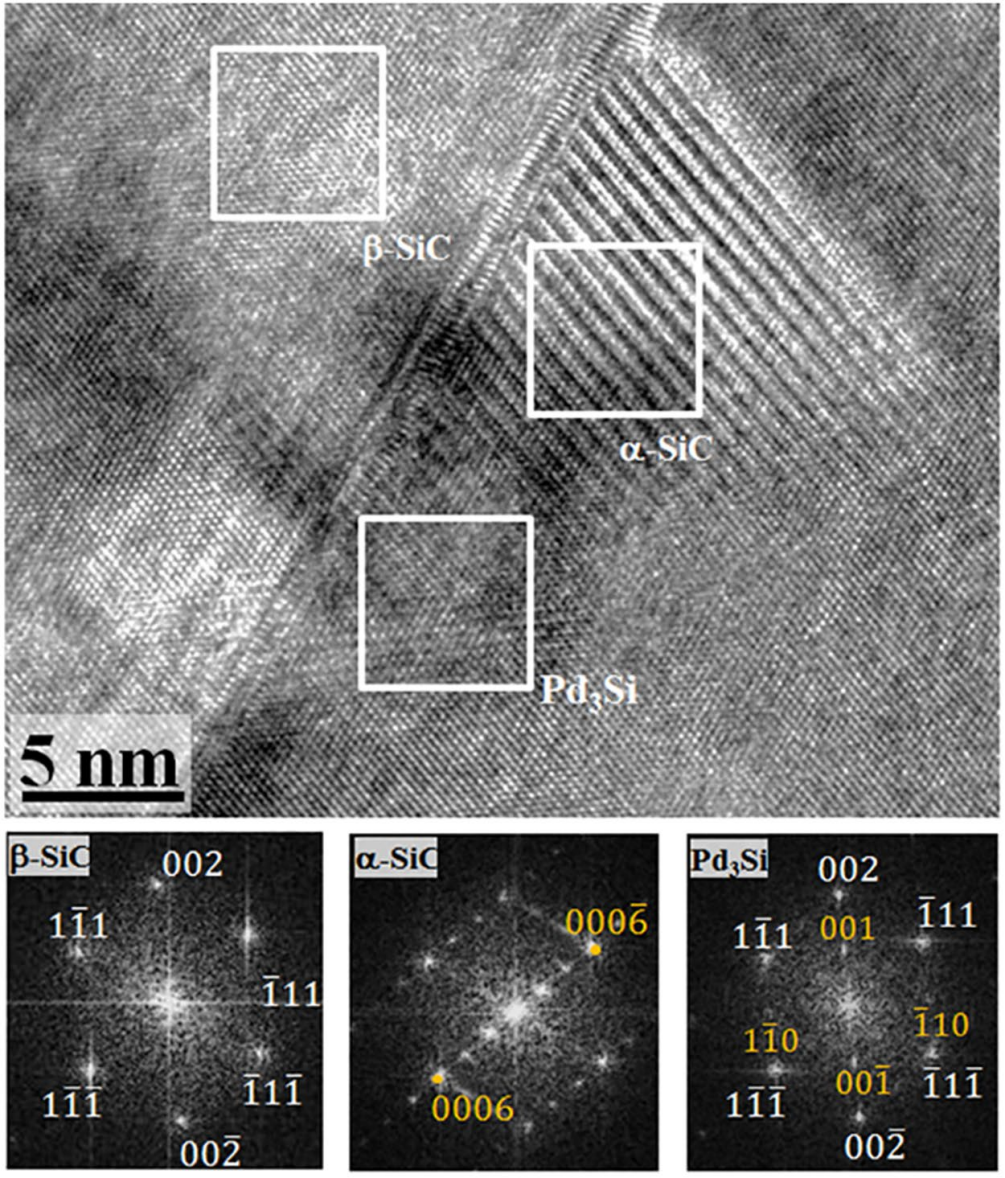
Structural changes within a partially transformed α-SiC precipitate. A HRTEM image along the [110] zone axis of the β-SiC matrix captures an intermediate stage of transition of α-SiC into a Pd silicide, along a Frank loop. The FFT of α-SiC shows the expected {111}_β_ || {0001}α orientation. The FFT of the Pd-rich region reveals the L1_2_ ordered structure of the Pd_3_Si.

The structural analysis of such a semi-transformed region in β-SiC is shown in Fig. 4. The FFT of the β-SiC matrix shows the characteristic diffraction pattern of cubic-SiC along the [110] zone axis. The FFT corresponding to the top part of the structure nucleated at a Frank loop indicates a 6 H variant of SiC, and the expected α–β orientation relationship, {111}_β_ ||{0001}_α_, is observed. The FFT of the bottom part of the structure shows the superlattice spots, apart from the principal β-SiC spots, corresponding to a primitive L1_2_ cubic structure.

A comparison of neutron irradiated TRISO fuel with out-of-pile experiments, as reported by van Rooyen *et al*.^30^, suggests that the out of pile experiments do not simulate the relevant diffusion coefficient of fission products in SiC. Although Pd silicide formation in SiC has also been observed in other type of irradiations such as swift heavy ions (SHI)^29^, there is no microscopy data available that can ensure the applicability of SHI to induce such nanostructuring in polymorphic ceramics. The comparison of microstructure under neutron and proton irradiation reports that proton irradiation can likely reproduce the microstructure evolution of neutron irradiation since the average size and number density of dislocation loops and voids are similar^31^. Hence, similar nanostructuring remains a possibility in various polymorphic ceramics such as ZrO_2_^32^ and ZrC^33^ by various irradiation sources.

In conclusion, the present study reports a heterogeneous nucleation process in a simple polymorphic crystalline solid that involves two discrete reconstructive and diffusive transformations in a sequence. The phase formed upon the irradiation-induced reconstructive transformation subsequently acts as a surrogate phase and facilitates its reaction with a fission product that is transported in the microstructure via linear and planar defects. The fission product precipitates adopt the exact morphology of the parent phase without movement of any phase boundary and bypass the activation energy otherwise required for its nucleation. These exemplary results challenge the conventional wisdom of precipitation in a nuclear reactor environment. They provide a new view of irradiation-induced nanostructuring in ceramics for advanced applications.

## Methods

TRISO coated nuclear fuel was fabricated under standard conditions and subjected to irradiation under the AGR-1 and AGR-2 experimental programs in the Advanced Test Reactor at Idaho National Laboratory. The irradiation conditions for these two programs are reported in Table 1. Samples for TEM were prepared by a dual-beam Quanta 3D focused ion beam instrument. STEM and conventional TEM analysis were conducted on an FEI Tecnai F30 microscope operated at 300 kV. STEM images were obtained using different camera focal lengths, ranging from 80 mm to 4.5 m, for better visualization of nanoscale features in β-SiC. Chemical analyses on TEM samples were carried out using the EDAX^TM^ energy dispersive spectroscopy (EDS) system. Gatan Digital micrograph and TIA^TM^ (TEM imaging and analysis) software were used for post-processing of TEM data. The simulations of diffraction patterns were carried out using JEMS^TM^ software.

**Table 1:** Irradiation parameters of TRISO fuel in AGR-1 and AGR-2 experiments.

Parameter	AGR-1	AGR-2
% FIMA average burnup	11.3	10.8
Time-averaged maximum temperature (°C)	1,144	1,335
Time-averaged volume average temperature (°C)	1,070	1,261
Approximate time at temperature (days)	620.2	559
Approximate fast fluence (n/cm^2^)	2.38 × 10^21^	3.0 × 10^21^
Fuel type	Baseline^34^	Variant 3^35^

### Data availability statement

The datasets generated during the current study are available from the corresponding author on reasonable request.

## Electronic supplementary material

Supplementary Information
