# Supplementary material for: A Novel Dual-Step Nucleation Pathway in Crystalline Solids under Neutron Irradiation
Source: Sci Rep. 2018 Jan 8;8:98. doi: 10.1038/s41598-017-18548-8 (PMC5758828; doi:10.1038/s41598-017-18548-8)
Supplement: Supplementary file 1 — Supplementary Information [file 41598_2017_18548_MOESM1_ESM.pdf]

Supplementary Materials for

A Novel Dual-Step Nucleation Pathway in Crystalline Solids under Neutron Irradiation

Subhashish Meher[1*], Isabella J. van Rooyen[2], and Thomas M. Lillo[1]

[1]Materials Science and Engineering Department, Idaho National Laboratory, Idaho Falls, ID, 83415, USA.

[2]Fuel Design and Development Department, Idaho National Laboratory, Idaho Falls, ID, 83415, USA.

*Correspondence to: subhashish.meher@inl.gov.

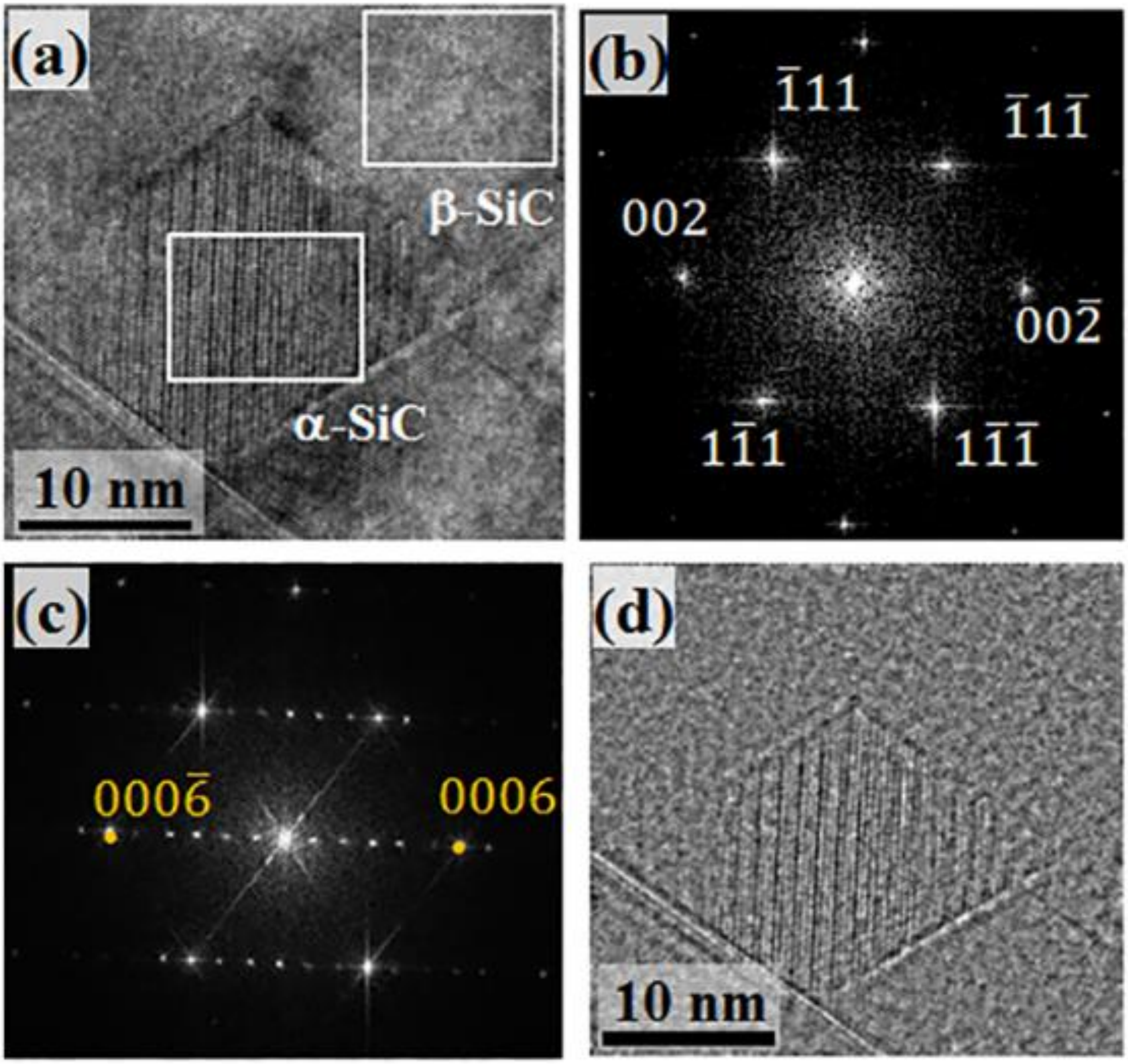

Figure S1. Unexpected crystallographic orientation of neutron irradiation-induced α-SiC precipitates with β-SiC. (a) A HRTEM micrograph of β-SiC along the [110] zone axis shows a α-SiC precipitate that lies on a Frank loop. b) A FFT of the β-SiC showing only the principal diffraction spots of a cubic SiC structure. c) A FFT of the α-SiC showing the diffraction spots corresponding to the 6H variant. The orientation relation as found here, $\{002\}_{\beta} \parallel \{0001\}_{\alpha}$, can occur if the basal plane of α-SiC is rotated to keep its $\langle 0001\rangle_{\alpha}$ parallel to $\langle 002\rangle_{\beta}$ of matrix β-SiC. d) An inverse FFT obtained by masking the spots corresponding to β-SiC shows the crystallography of α-SiC and the Frank loop.

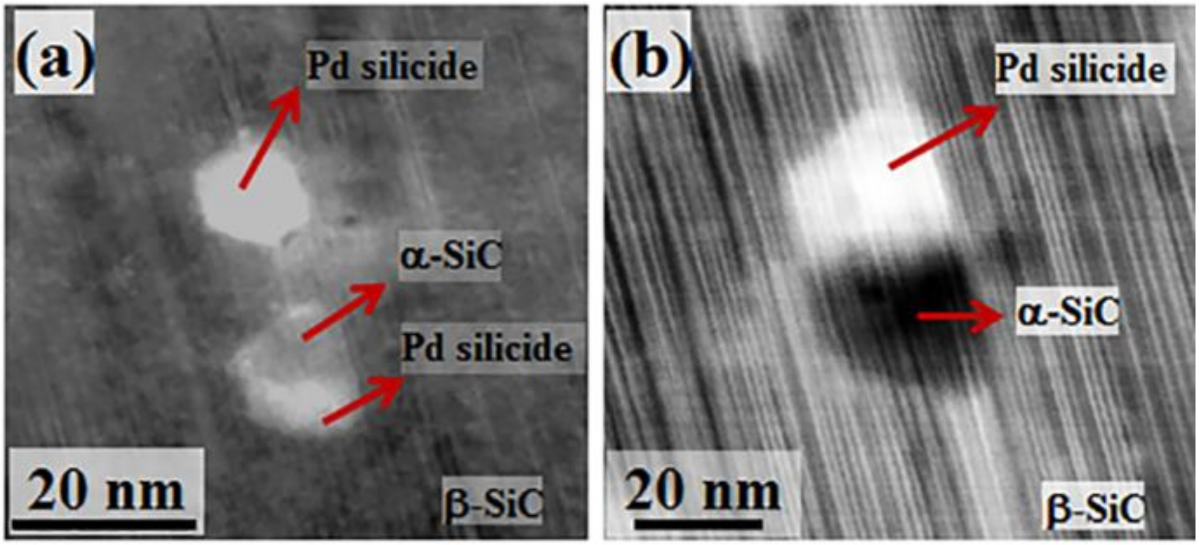

Figure S2. Precipitation of nanoscale polygonal precipitates at stacking faults in the β-SiC layer of a TRISO fuel particle in the AGR-2 experiment. Both (a) and (b) show semi-transformed precipitates.
